# Improvement of survival rates in the last decade in Thai childhood-onset systemic lupus erythematosus

**DOI:** 10.1186/s12969-018-0274-5

**Published:** 2018-09-29

**Authors:** Pondtip Jongvilaikasem, Edward B. McNeil, Pornsak Dissaneewate, Prayong Vachvanichsanong

**Affiliations:** 1 Department of Pediatrics, Faculty of Medicine, Prince of Songkla University, Hat Yai, Songkhla 90110 Thailand; 2 Epidemiology Unit, Faculty of Medicine, Prince of Songkla University, Hat Yai, Songkhla Thailand

**Keywords:** Childhood-onset, Survival rates, Systemic lupus erythematosus, Thai

## Abstract

**Background:**

Morbidity and mortality in childhood onset systemic lupus erythematosus (SLE) is more severe than adult onset SLE. Long-term follow up is needed to determine the prognosis. The objectives of this study are to describe the mortality of childhood SLE in a single tertiary care centre over three decades, compare trends in survival over time, and determine predictors for survival.

**Methods:**

We retrospectively reviewed the medical records of children aged < 18 years who were diagnosed with SLE at the Department of Pediatrics, Songklanagarind Hospital, Thailand, from 1985 to 2016.

**Results:**

There were 331 children (272 girls, 59 boys) with a mean age at presentation of 11.5 ± 2.6 years. The mean follow-up duration was 7.0 ± 5.0 (range 1–28) years, 77 children (23.3%) died, 28.6% within the first year after diagnosis.

The overall mortality rate was 3.3 per 100 person-years. Survival rates at 1, 5 and 10 years were 93.4%, 83.1% and 72.6%, respectively. Ten-year survival rates for the children diagnosed in the decades 1985–1996, 1997–2006 and 2007–2016 were 67.4%, 63.4% and 82.8%, respectively (*p* < 0.001).

Boys had worse survival than girls (hazard ratio = 2.3, 95% CI: 1.4–3.7) even after adjusting for decade of diagnosis. Lupus nephritis (LN) class IV had similar survival compared to LN classes II/III/V combined (hazard ratio = 1.0, 95% CI: 0.6–1.7).

**Conclusion:**

In our setting, the survival rate of childhood onset SLE has improved during the past 10 years, but mortality is still high compared to developed countries, particularly in boys.

## What’s known on this subject

The mortality and survival rates of childhood-onset systemic lupus erythematosus in developed countries have improved in the last decade, and 10-year survival has improved to greater than 90%.

## What this study adds

This study was conducted in a single center with the largest number of Thai childhood onset systemic lupus erythematosus cases and a 30-year follow-up. Although the mortality and survival rates have improved over time, Thailand still has lower rates than developed countries.

## Background

Systemic lupus erythematosus (SLE) is one of the most common and severe connective tissue diseases in children. Although it is not a very common disease in general practice, it is a diverse disease and causes damage with devastating sequelae [1]. By definition, SLE is a multi-system autoimmune disease. The severity varies widely depending on the affected organ or organs, with renal and neuropsychiatric involvement being more serious than involvement of other organs [2, 3]. Patients with SLE require specialist medical care depending on the major organ involvement.

Childhood-onset SLE (cSLE) constitutes between 10 and 31% of all SLE cases but has a higher severity and poorer outcome than adult onset SLE (aSLE) [3–6].

cSLE can be a critical disease and is a chronic condition with control of the disease attainable with or without treatment or disease flares. In the 1950s the survival rate was only 50% [7], but after the introduction of steroids in 1970 survival rates dramatically improved, although it was still a debilitating disease [8]. Survival rates continued to improve following the introduction of new effective immunosuppressive drugs during the 1980s and 1990s,which also lowered side effects, due to advances in medical technology and knowledge, and more intensive management such as renal replacement therapy [8].

With more and more new treatment modalities, cSLE survival rates have continued to improve over the recent decades, and 10-year survival rates from developed countries are now as high as 90% and as high as 100% at 5 years [2, 9–11].

Ethnicity has been shown to influence SLE outcomes with African-American, Hispanic and Asian populations having a higher risk of mortality and end-stage renal disease (ESRD) than Caucasians [10, 12]. Another study found that in children with SLE, race was associated with admissions to an ICU, ESRD and death, where African-Americans had an increased risk of ICU admissions, and African-Americans and Hispanics had an increased risk of ESRD and death [13].

Studies of mortality rates in cSLE in developing countries are required to evaluate the results of available modern therapies and determine the optimal therapy modalities in limited resource settings.

There have been few studies available to investigate long-term outcomes in a large single group of children. In addition, due to the wide severity of SLE, outcomes are difficult to compare between studies [1].

## Objective

To describe the mortality of cSLE in a single tertiary care center in southern Thailand over a 30-year period, compare trends in survival over time, determine predictors for survival, and compare our results with current studies from other countries.

## Methods

We retrospectively reviewed the medical records of children aged < 18 years who were diagnosed with SLE and presented to the Department of Pediatrics, Songklanagarind Hospital, southern Thailand from February 1985 to August 2016. All the children were followed up for at least one year. The duration of follow-up was defined as from the time of SLE diagnosis until the patient was last seen or contacted by the hospital. All patients met the American College of Rheumatology revised criteria for the classification of SLE [14]. Age at diagnosis of SLE in patients who were referred to our institution was determined from the date of their first presentation. This study was approved by Ethics Committee of the Faculty of Medicine, Prince of Songkla University, Thailand.

The morphological classification of lupus nephritis (LN) followed the World Health Organization (WHO) system [15]. We defined class I as normal glomeruli and classes II-VI as mesangial proliferative, focal segmental, diffuse proliferative, membranous and advanced sclerosing glomerulonephritis, respectively. The most severe LN class detected from the renal biopsies in patients who had repeated biopsies was used for analysis.

We divided the patients into three groups based on the period of diagnosis: 1985–1996, 1997–2006 and 2007–2016. Gender-specific mortality and survival rates were compared across these three groups, and survival rates compared for different LN classifications.

Statistical analysis was performed using R software, version 3.4.0 [16].

Means and standard deviations (sd) were used to present the results descriptively. Pearson’s Chi-squared test (or Fisher’s exact test where appropriate) was used for testing associations between categorical variables. The Wilcoxon rank sum test was used to compare non-normally distributed continuous variables. Kaplan-Meier survival curves were generated to compare survival rates, and the Peto & Peto modification of the Gehan-Wilcoxon test was used to compare survival differences among sub-groups (this test gives more weight to early outcomes compared to the logrank test). Multivariate analysis using Cox proportional hazards regression was used to determine the risk factors for overall survival. *P* values less than 0.05 were considered statistically significant.

## Results

There were 331 children with cSLE treated in our institute during the 30-year period, 272 girls and 59 boys. The mean (sd) age at presentation to our hospital was 11.5 ± 2.6 years. (range 2.3–18.0) (girls 11.7 ± 2.6, boys 11.1 ± 2.6, *p* = 0.13). During a mean (sd) follow-up duration of 7.0 ± 5.0 years (girls 7.2 ± 5.1, boys 6.0 ± 4.8, *p* = 0.1), 77 (23.9%) died, 22 (28.6%) within the first year, 8 (10.4%) in the second year, 8 (10.4%) in the third year, and 39 (50.6%) after the 3^rd^ year. 36 children were lost to follow-up and 105 were referred to adult services after turning 18. Among the 36 children lost to follow-up, the mean (sd) follow-up time was 7.6 ± 5.1 years (range 1.4–26 years) (attempts were made to contact these children without success).

Renal biopsy was performed in 256 children (75.8%) and showed LN classes I, II, III, IV, and V in 11, 106, 9, 102, and 23 children, respectively. In five the tissue was not adequate for evaluation. The remaining 75 did not have renal biopsy performed due to no manifestation of renal involvement, custodian refusal, or death.

**Fig. 1:**
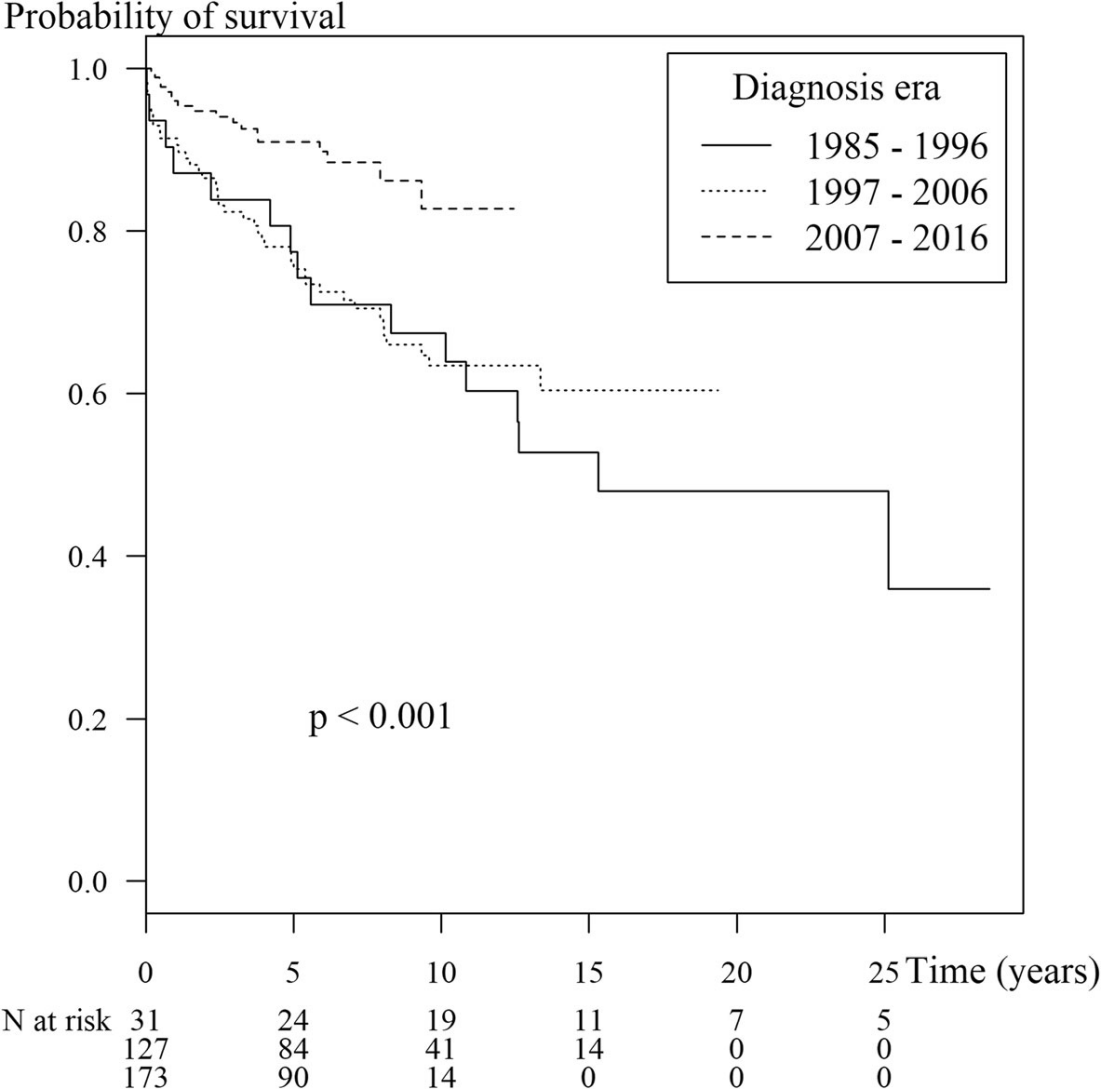
Kaplan-Meier survival curve showing survival by year of diagnosis in 331 childhood-onset SLE cases and number of patients at risk during each time period

**Fig. 2:**
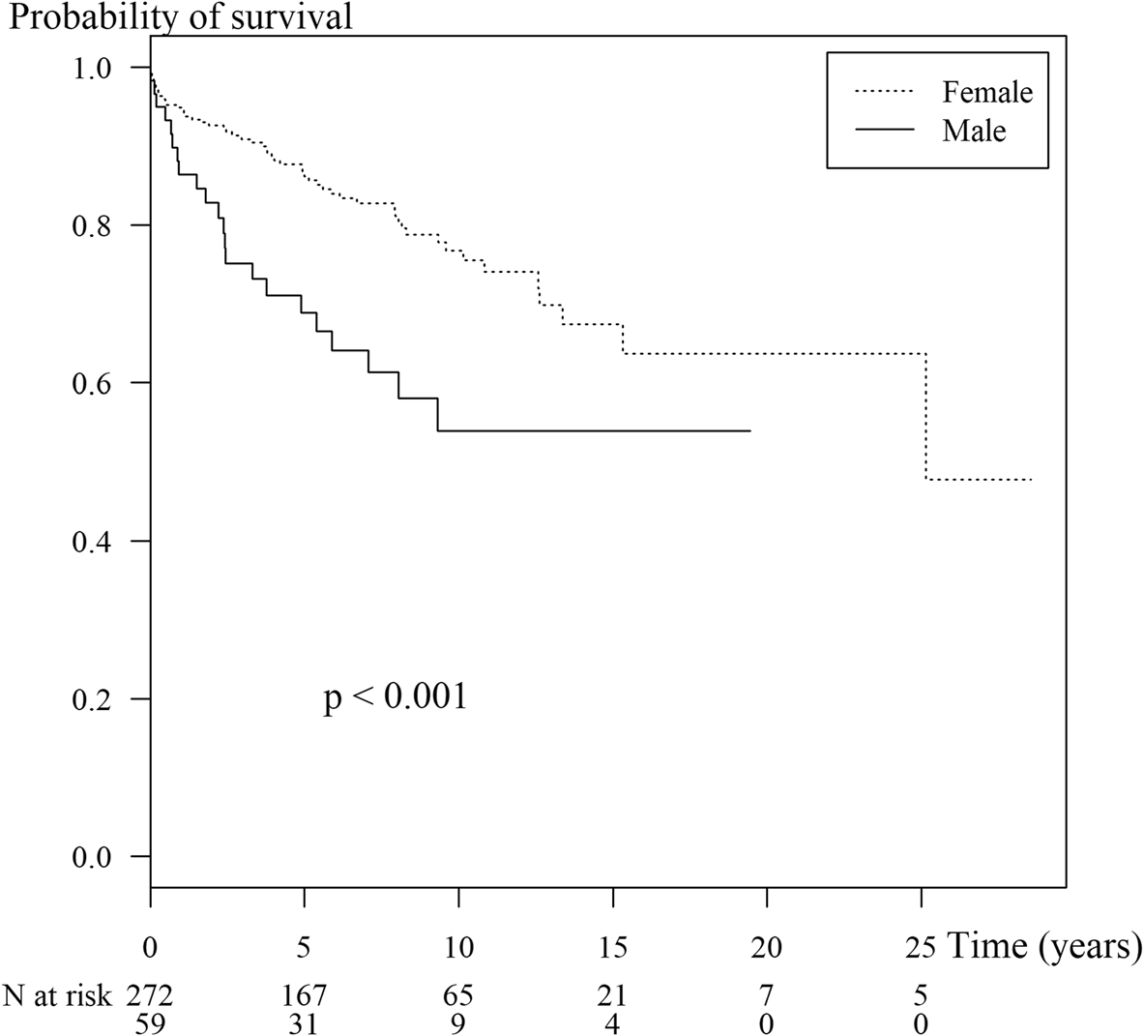
Kaplan-Meier survival curve showing survival in 331 childhood-onset SLE cases by sex and number of patients at risk during each time period. The vertical ticks represent censored observations

The overall mortality rate was 23.3% or 3.3 per 100 patient years. The overall cumulative probabilities of survival after diagnosis at 1, 5 and 10 years were 93.4%, 83.1% and 72.6%, respectively. As shown in Figure 1, the Ten-year survival rates for children diagnosed in the decades 1985–1996, 1997–2006 and 2007–2016 were 67.4%, 63.4% and 82.8%, respectively (*p* < 0.001). Boys had worse survival than girls (*p* = 0.03, Fig. 2).

Table 1 shows the results of the multivariate Cox regression model. Gender and decade of diagnosis were the only significant factors associated with survival. Boys had a higher hazard of dying (HR = 2.0, 95% CI = 1.22–3.35) compared to girls while children diagnosed in the most recent decade had a significantly lower hazard of dying (HR = 0.35, 95% CI = 0.17–0.71) compared to children diagnosed in the first era. There were no differences in survival among the LN classes. Further models comparing LN class IV to LN classes I, II, III and V separately and combined also found no significant difference in survival.

**Table 1:** Multivariate Cox regression results for survival of childhood-onset SLE

	Hazard Ratio (95% CI)	*P*-value (Wald’s test)	*P*-value (LR-test)
Gender: Male vs Female	2.02 (1.22–3.35)	0.006	0.009
Decade of diagnosis: ref. = 1985–1996			0.002
1997–2006	0.73 (0.43–1.25)	0.25	
2007–2016	0.31 (0.15–0.63)	0.001	
LN class: ref. = Class I			0.58
Class II	2.48 (0.33–18.8)	0.38	
Class III	4.70 (0.51–43.0)	0.17	
Class IV	2.59 (0.35–19.0)	0.35	
Class V	2.12 (0.24–19.1)	0.50	
Not done/mixed result	3.48 (0.46–26.5)	0.23	

*CI* Confidence interval, *LR-test* Likelihood ratio test, ref.: reference group

**Fig. 3:**
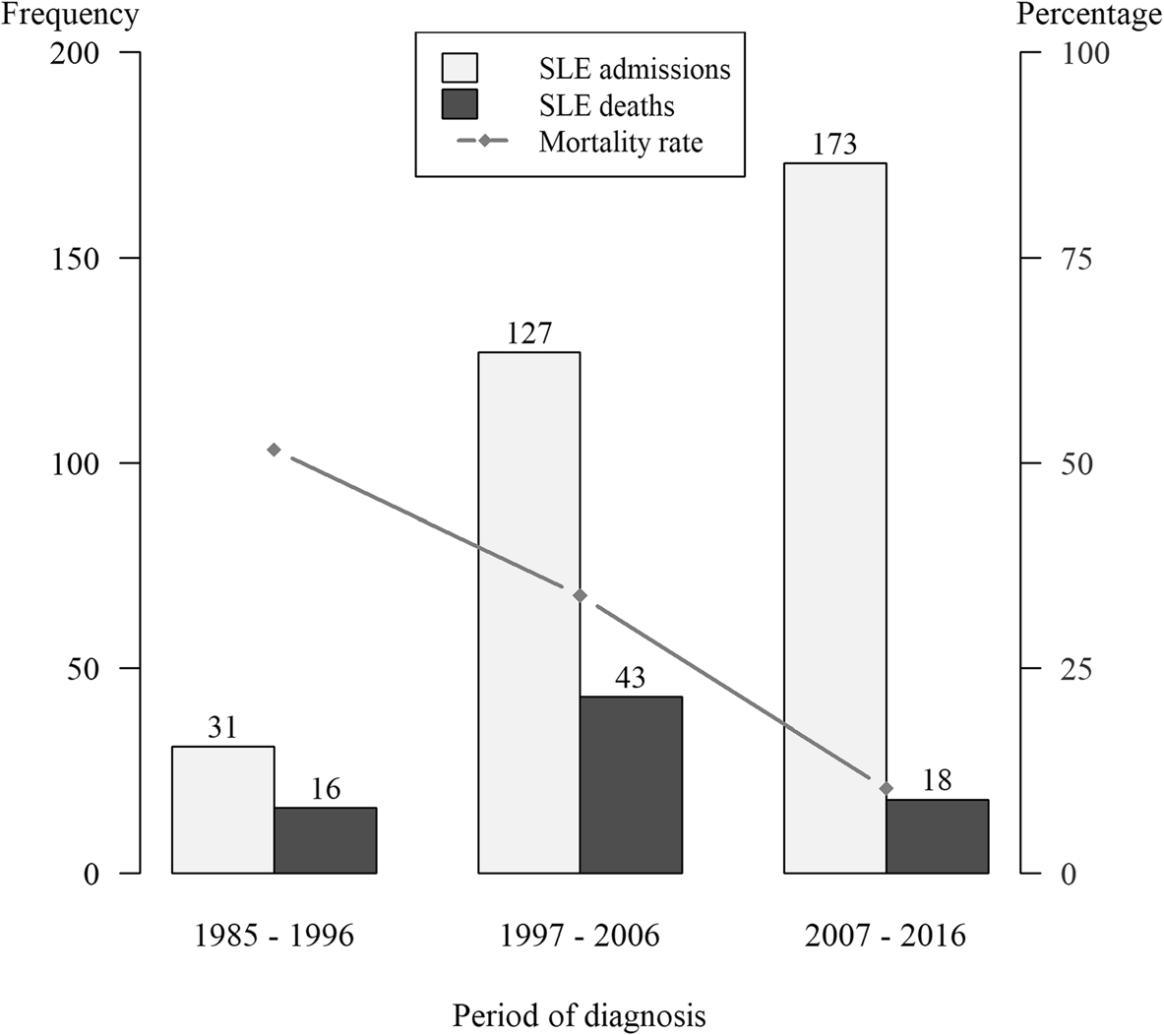
Comparison of SLE diagnosis and SLE-related death and mortality in the 3 study periods. Frequency (left vertical axis) refers to number of SLE admissions and deaths while the percentage (right vertical axis) refers to mortality rates

Figure 3 shows the trend in mortality rates over the 32-year period. The rate decreased from 52% during 1985–1996 to 34% during 1997–2006 and further to 10% during 2007–2016.

## Discussion

Our study describes the mortality and survival rates of cSLE from a pediatric nephrologist’s point of view in the setting of a national university hospital which provides tertiary medical care. Severe patients are referred to this hospital from local primary and secondary healthcare facilities in all 14 provinces of southern Thailand. Therefore, our sample probably contained more severe patients, particularly those with severe renal involvement, and may not reflect the actual severity and mortality rates of cSLE in general in southern Thailand.

This study was conducted in a comparatively large number of patients from a single center with long-term follow up. Although SLE is a disease common in child-bearing females due to its association with female hormones, the youngest patient in our study was only 2.3 years old. Our male:female ratio was 1:4.6 which was similar to other studies in cSLE [10, 17]. A study from Iran found that the male:female ratio was lower for cSLE compared to aSLE (1:3.3 vs. 1:8.4, respectively) [3].

The survival rates in our study improved during the last decade, a result consistent with other studies in adults and children from developed countries [10, 18]. However, the improvements in Thailand are still behind those from developed countries. To date, there are few studies on long-term outcomes including mortality rates in cSLE. Such comparisons of outcomes, however, are quite limited due to differences in study settings, disease severity, ethnicity, and treatment regimens.

Our study was conducted in a situation that is more likely to have poor outcomes due to the most severe cases being referred, usually with renal involvement and from a setting with no renal replacement therapy. In addition, we could not compare ethnicities in this study since all the children were Thai.

Table 1 shows that in our study only gender and decade of diagnosis were independent factors associated with survival. Males (HR = 2.3) had a higher risk of death compared to females and children diagnosed in the most recent decade had a 2.5 times more favorable prognosis compared to children diagnosed before 2006. LN severity was not associated with survival, which was consistent with our previous study [19]. In contrast, a study by Abujam et al. found that the proportion of SLE cases with renal involvement determined by azotemia, hypertension, hematuria and proteinuria was significantly higher in those who died than those who survived [20].

However, about 20% of our cases did not have a renal biopsy performed for various reasons and the number of patients with LN classes II and IV were similar while other studies have found that LN class IV predominated [2, 4, 5, 21, 22].

In the setting of a rheumatology clinic, a study compared 31 juvenile-onset SLE (jSLE, age 13–18 years) with 48 aSLE (age 19–50 years) who were followed up at for at least one year, and found that renal and neuropsychiatric manifestations in jSLE were greater than in aSLE [23]. Additionally, the same study found that SLE-related hospitalizations in jSLE were greater than in aSLE (67.7% vs. 37.5%), and the mortality rate of jSLE was almost two times higher than in aSLE (19.4% vs 10.4%), however this difference was not statistically significant, probably due to the small sample size.

Watson et al. [12] reported from a pediatric rheumatology and nephrology clinic in 198 jSLE cases where the majority were Caucasians from the United Kingdom and males were significantly younger than females. The overall male:female ratio was 1:5.6, but among those aged < 10 years the ratio was 1:2.7. Additionally, in their cohort study they found that non-Caucasians had a greater risk of SLE than Caucasian patients. In our study the ages were not significantly different between males and females.

Table 2 shows a comparison of mortality and survival rates among cSLE cases from various studies from different countries.

**Table 2:** Comparison of patient survival among various studies of childhood-onset SLE

Author(s), (publication year)	Country of Study	Duration of study	N	Follow-up period mean ± sd (range) (yr)	M:F	Mortality rate (%)	Survival rate			Remark
							1 year	5 year	10 year	
Present study	Thailand	1985–2016	331	7.7 ± 4.9 (1–28)	1:5	24	93	82	72	Pediatric Nephrology Clinic
		*1985–1996*	*52*	*14.7 ± 6.9 (1–28)*	*1:4*	*52*	*89*	*66*	*62*	
		*1997–2006*	*136*	*9.4 ± 3.9 (1–18)*	*1:5*	*34*	*93*	*79*	*69*	
		*2007–2016*	*143*	*5.1 ± 2.5 (1–10)*	*1:5*	*10*	*96*	*91*	*89*	
Baqi, 1996 [17]	USA	1965–1992	56	5.3 (6 mo-20)	1:5	16	–	44	29	64% Black, 50% ESRD
Yang, 1994 [25]	Taiwan	1979–1991	167	4.9 (1–13)	1:11	9	–	91	–	All LN, 10 ESRD
Hagelberg, 2002 [10]	Canada	1984–1991	67	11.0 (5–19)	1:4	6	–	97	95	All LN
Wong, 2006 [9]	Hong Kong	1990–2003	128	5.8 ± 3.6 (−)	1:15	4	–	95	92	40% renal involvement
Lee, 2013 [2]	Taiwan	1990–2012	189	6.9 ± 4.6 (0–22)	1:7	7	–	93	90^a^	Pediatric Rheumatology Clinic, LN 52%
Abujam, 2016 [20]	India	1991–2013	122	4.8 ± 4.5 (3 mo-20)	1:3	20	88	78	71	General Pediatric Center
Fatami 2017 [3]	Iran	1992–2013	180	–	1:3	11		91	87	
Al-Mayouf, 2008 [24]	Saudi Arabia	1995–2007	89	5.1 (−)	1:6	9		91	–	Most affected organ: neuropsychiatric
Tavangar-Rad 2014 [26]	Iran	2004–2010	120	4.7 ± 2.7 (1–10)	1:3	10	97	89	–	Pediatric Rheumatology Clinic, LN 60%

*ESRD* end stage renal disease, *LN* lupus nephritis

^a^10–20 year survival rates

Lee, et al. [2] reported survival rates at 5 and 10 years in 189 cSLE cases with 22 years follow-up from a pediatric rheumatology clinic in Taiwan of 93.4% and 89.6%, respectively. This study was similar to our study in terms of period of study, duration of follow-up, and race, however the male:female ratio was slightly higher, and the percentage of patients with lupus nephritis was only 52%. They showed better survival and mortality rates and found that survival in cSLE with and without LN were not significantly different.

Our study found that females had a better outcome than males, a result similar to one study [3]. but contrasting with two others; a cSLE study [24] and a mixed onset SLE study which included both cSLE and aSLE [6].

A study from India during a similar study period and setting but in a smaller number of patients (*n* = 122) and having a slightly shorter duration of follow-up (range 3 months-20 years), reported that mortality and survival rates were similar to our study and survival rates similarly improved over time [20].

A study from Saudi Arabia also achieved a 91% survival rate at 5 years in cSLE with neuropsychiatric SLE being the most common manifestation. Additionally, their mortality rate was significantly higher among children aged < 5 years compared with those aged > 5 years (25% vs. 6.5%, *p* = 0.03) [24].

Two cSLE studies from a rheumatology clinic in Taiwan and a nephrology clinic in Hong Kong both reported 10-year survival rates of around 90%. The renal involvement was low in both studies (40% and 52%) and the male:female ratios were both high (1:7 and 1:15), which may explain the high survival rates since females are known to have a better survival rate [2, 9]. Two studies reported that the nature of cSLE with LN was poorer than cSLE without LN [2, 20]. Our study was conducted in a Pediatric Nephrology Clinic which had more than 70% renal involvement based on histopathology, which may also indicate that the more the renal involvement the poorer the survival rate. Additionally, our study had a higher proportion of males (male:female ratio = 1:5), and all of these factors taken together would result in a poorer survival rate.

The most impressive study in terms of survival rate was a study from Taiwan in 167 cSLE cases all with LN and 10 had ESRD, which found a 5-year survival rate of 91% in the 1990s. [25]. This high survival rate in a group of children with severe disease could have been due to the low proportion of males in the study (male:female ratio = 1:11).

Although the mortality and survival rates in our institute improved over time, a result consistent with other studies, our study is incomparable since mortality rates are influenced by many factors such as gender, age, race/ethnicity, setting, treatment modality, year of diagnosis, major organ involvement and severity of disease.

In general, therapy protocols significantly affect morbidity and mortality. However, in this retrospective study the comparison of treatment protocols in the three decades is not possible due to the nature of SLE disease which has a multi-organ involvement, therefore therapy was absolutely adjusted by the clinician following the major organ involvement and disease activity (flare). In fact, SLE treatment has advanced over time due to new immunosuppressive drugs, antibiotics and palliative therapy, particularly renal replacement therapy (RRT), which altogether results in improved outcomes.

In our institute, induction therapies include prednisolone, pulse methylprednisolone, intravenous cyclophosphamide, and/or mycophenolate mofetil. Maintenance therapy includes prednisolone, oral cyclophosphamide, mycophenolate mofetil, and/or azathioprine, and the duration therapy depends on disease activity.

The significant improvement of survival rates across the time periods in our study undoubtedly reflect the continually increasing availability and quality of health care in Thailand. Nowadays, patients are seeking medical care earlier than in previous years, due to better education, and they also show better compliance with all forms of treatment than in earlier years. Primary care physicians can also now make earlier diagnoses of SLE and its complications and refer needful patients to a health care center, such as our institute, where more effective immunosuppressive drugs, antibiotics for infection, and other palliative care options such as RRT are available.

It is crucial to identify the mortality and survival rates of children with SLE in order to understand the seriousness of the situation. Determining the associated factors is necessary to improve the survival of these vulnerable patients. Moreover this information will reflect the nature of the disease and the ability to provide adequate treatment. A multidisciplinary effort should be implemented to achieve optimal response to treatment to rescue the patient during the critical period and to prevent and minimize organ damage in the long-term. Ultimately mortality and survival rates will be improved.

## Conclusion

In our setting, the survival rate of childhood onset SLE has improved notably over the last three decades, but mortality is still high compared to developed countries, particularly in boys. Most deaths occur within the first year following presentation. The severity of lupus nephritis does not determine survival.

## Abbreviations

aSLE: Adult-onset systemic lupus erythematosus
cSLE: Childhood-onset systemic lupus erythematosus
ESRD: End-stage renal disease
HR: Hazard ratio
jSLE: Juvenile-onset systemic lupus erythematosus
LN: Lupus nephritis
RRT: Renal replacement therapy
sd: Standard deviation
SLE: Systemic lupus erythematosus
WHO: World Health Organization
